# Construction and Application of a Tactile Somatosensory Comfort Model for Scrubbing Tasks

**DOI:** 10.3390/biomimetics11040237

**Published:** 2026-04-02

**Authors:** Peng Xu, Chang Zhai, Yipeng Xiao, Leigang Zhang, Hongliu Yu

**Affiliations:** 1 Institute of Rehabilitation Engineering and Technology, University of Shanghai for Science and Technology, Shanghai 200093, China; 201560120@st.usst.edu.cn (P.X.); 232322266@st.usst.edu.cn (C.Z.); 243352470@st.usst.edu.cn (Y.X.); 2 Shanghai Engineering Research Center of Assistive Devices, Shanghai 200093, China; 3 School of Mechatronic Engineering and Automation, Shanghai University, Shanghai 200444, China

**Keywords:** tactile somatosensory comfort, stevens’ law, energy transfer function, scrubbing parameter optimization, heart rate variability (HRV)

## Abstract

Tactile somatosensory comfort is a critical factor in ergonomics research, particularly in designing assistive robots for geriatric care. Despite its importance, existing studies lack comprehensive comfort models tailored for optimizing system control in such applications. This study addresses this gap by introducing the first derivation of a tactile somatosensory comfort model that integrates Stevens’ law with the energy transfer function, establishing a link between physical stimuli and psychological responses. Through experimental data collection and parameter fitting, a quantitative relationship between comfort and psychological responses was established, facilitating the development of a novel optimal control model. The model parameters were fitted using the Physics-Informed Neural Networks (PINNs) algorithm, while the optimal scrubbing parameters for force (1.68 N) and velocity (36.47 mm/s) were determined via the Particle Swarm Optimization (PSO) algorithm. Validation experiments involving 20 participants, which monitored physiological parameters such as heart rate variability (HRV), confirmed the model’s effectiveness in enhancing comfort while ensuring robustness and generalizability. These findings contribute a novel theoretical framework for modelling and applying tactile somatosensory comfort, providing valuable insights for future research and development.

## 1. Introduction

The sense of touch is a fundamental means by which humans engage with their surroundings, with the skin functioning as the primary sensory interface [1]. Mechanically stimulated skin undergoes mechanical deformations, including compression and stretching, activating nerve endings that generate electrical signals transmitted to the cerebral cortex, resulting in sensation [2]. David Julius and Ardem Patapoutian’s Nobel Prize-winning research (2021) elucidates the mechanisms of temperature and touch perception, emphasizing the role of human receptors in generating sensations like vibration, pressure, and temperature [3]. The skin, as the body’s largest sensory organ, plays a pivotal role in environmental perception, particularly through physical interactions, where signals are shaped by factors like contact force, motion dynamics, temperature, and humidity [4,5]. These biological sensing and response mechanisms have also inspired the emerging field of biomimetics, where engineering systems attempt to replicate the structure and function of human skin to achieve more natural and effective tactile interaction [6]. Despite its critical role, the skin’s complex mechanical and physiological properties present significant challenges to understanding friction sensing mechanisms [7,8].

The frictional properties of skin during sliding contact are influenced by input variables such as the mechanical and geometric properties of the contact friction pair [9], the lubricating medium, applied force, and environmental conditions [10,11]. Output variables include shear forces, skin deformation, and perceptual and emotional responses, including sensations of comfort and tension [12,13]. This research offers promising avenues for improving tactile comfort in assistive robotic systems. In particular, biomimetic approaches in robotic skin design and tactile interaction aim to reproduce human-like frictional perception and response, further emphasizing the importance of quantitatively modeling the relationship between physical stimuli and perceived comfort [14]. For example, Schwartz’s slip hypothesis provides a framework for understanding tactile perception by describing tactile information encoding via frictional slipping. [15] However, its limited empirical validation from the human tactile system restricts its generalizability. Similarly, studies on tribological interactions, such as those involving Braille dot patterns or friction on smooth and rough surfaces, have yielded valuable insights but frequently depend on oversimplified assumptions or specific contexts, limiting their broader applicability [16,17].

Subjective sensory quantification has become a complementary approach for studying skin friction characteristics. For instance, integrating psychophysical assessments with tribological analyses has deepened our understanding of tactile properties, including roughness and smoothness [18]. However, these methods often encounter limitations, including significant inter-individual variability in subjective perception, difficulty in establishing quantitative relationships between physical stimuli and perceptual or neural responses, and challenges in integrating multi-modal data into a unified and predictive modeling framework [19]. Investigations into the influence of fluid environments on tactile perception further highlight the intricate interplay between subjective perceptions and tribological factors, though accurately replicating in vivo conditions continues to pose challenges [20]. From a biomimetic perspective, these limitations also hinder the development of artificial tactile systems that can faithfully reproduce human-like perception, underscoring the need for models that bridge physical interaction and perceptual response [21].

Comfort, a comprehensive metric of satisfaction stemming from both mental and physical perceptions, is influenced by numerous environmental and physiological factors [22]. In this study, comfort is defined as a multidimensional perceptual state resulting from the interaction between external physical stimuli and human sensory processing, encompassing both psychological experience and physiological response. It is quantitatively evaluated using subjective assessments (e.g., Visual Analog Scale, VAS) and objective physiological indicators (e.g., heart rate variability, HRV), providing a combined measure of perceived and physiological comfort. Understanding the mechanisms underlying skin friction perception is essential for developing reliable models of tactile somatosensory comfort [23]. Current research largely emphasizes applications such as textile friction and environmental conditions [24,25]. However, physical tactile comfort has become increasingly critical in evaluating technologies like care robots. This is particularly relevant in biomimetic robotics, where achieving human-like tactile interaction is essential for safe and comfortable human–robot interaction [26]. Recent studies using multi-channel frameworks that integrate surface topography, psychophysical assessments, tribological analyses, and EEG monitoring underscore the potential for advanced tactile perception models. Nonetheless, these approaches often face challenges related to complexity and data collection efficiency [27,28]. However, a significant gap remains in establishing a unified, quantitative framework that directly links physical interaction parameters (e.g., force and velocity) to subjective tactile comfort perception. Existing approaches either focus on physical modeling without perceptual interpretation or rely on subjective evaluation without predictive capability, limiting their applicability in control-oriented robotic systems. To address this gap, this study integrates an energy transfer function, which quantifies the physical stimulus, with Stevens’ psychophysical power law, which maps stimulus intensity to perceived sensation, thereby enabling a predictive and control-oriented tactile comfort model.

Given the non-linearity of the tactile body comfort model parameters, this study aims to establish a tactile body comfort model through data collection and regression analysis. The main contributions of this paper are as follows: (1) establishing a physics-psychophysics coupled framework that quantitatively links robot control variables to tactile comfort perception; (2) identifying model parameters through a physics-informed learning strategy that preserves the interpretability of the derived comfort model; and (3) constructing a closed-loop optimization method for scrubbing parameter selection based directly on predicted human comfort, followed by experimental validation using both subjective and physiological indicators.

## 2. Materials and Methods

### 2.1. Intelligent Bathing Robot System

**Figure 1:**
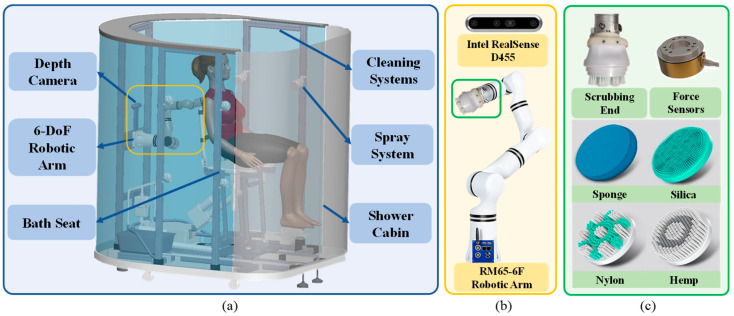
(**a**) Intelligent assisted bathing robot system, (**b**) assisted scrubbing system and (**c**) auxiliary scrub end.

To address the daily bathing needs of disabled elderly individuals and other people with disabilities, this paper presents the design of an intelligent assisted bathing robot system, as shown in Figure 1a. The system utilizes a modular design approach and comprises several key components: a bathing cabin, a bathing seat, a spraying system, a six-degree-of-freedom (6-DoF) bathing robotic arm, and a depth camera. This system is capable of safely and efficiently assisting users in transferring into the bathing cabin. Once inside, the robotic arm is designed to scrub all parts of the user’s body, ensuring a thorough and comfortable bathing experience. Besides, we introduce and summarize the mathematical notations that we used in this paper with Table 1.

#### 2.1.1. Scrubbing System

The bathing robot arm primarily consists of a 6-DoF serial robotic arm, a scrubbing end, and a depth camera, as shown in Figure 1b. The 6-DoF configuration was selected to provide sufficient kinematic flexibility for simultaneously controlling position and orientation, enabling the scrubbing end to adapt to the complex curvature of human body surfaces and maintain alignment with local surface normal during contact. A six-dimensional force sensor is installed at the connection between the robotic arm and the scrubbing end, enabling real-time detection and feedback of the contact force between the scrubbing end and the user’s body. This allows for closed-loop control of the contact force to ensure safety and comfort. Compared with lower-DoF configurations, which may be limited in handling non-planar surfaces, the 6-DoF robotic arm offers improved adaptability and interaction stability for human-robot contact tasks. Alternative configurations (e.g., redundant or compliant manipulators) are also promising and will be explored in future work. Additionally, the scrubbing end is available in various materials, including silicone, sponge, and nylon soft bristles, offering flexibility for different user needs. In this study, to ensure experimental consistency and eliminate the influence of varying material properties (e.g., friction coefficients and compliance), all validation experiments were conducted using a nylon brush head. Comparative analysis of different materials was not included in the current work and will be investigated in future studies. It features a quick-release and attachment mechanism, facilitating rapid replacement of the scrubbing end, as shown in Figure 1c. Both the robotic arm and scrubbing end are sealed to meet ingress protection (IP) 56 waterproof and dustproof standards. Meanwhile, the depth camera captures 3D coordinate information of the skin surface contour, ensuring the robot’s positioning accuracy during operation.

#### 2.1.2. Scrubbing Process

**Figure 2:**
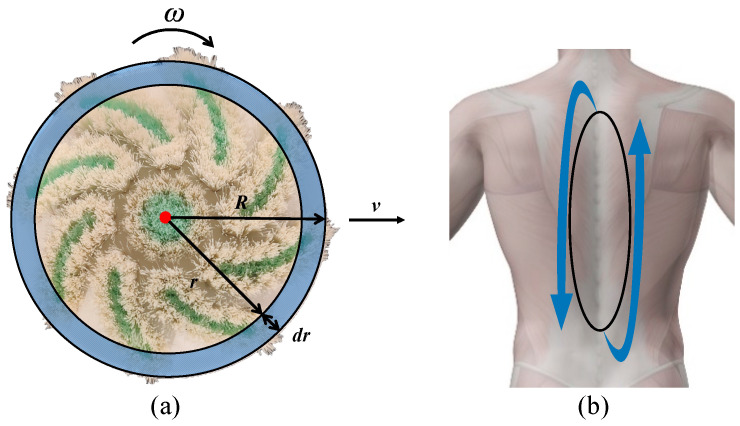
(**a**) Schematic of auxiliary scrub end movement and (**b**) scrubbing track.

During the scrubbing process, the bathing robotic arm achieves the scrubbing action by combining the self-rotating motion of the scrubbing end with its translational motion, as depicted in Figure 2a. This combined motion is designed to mimic typical caregiver-assisted bathing actions, where both rotational rubbing and linear wiping are involved. While the elderly remain stationary, the robotic arm collects pressure data between the scrubbing end and the skin surface through a six-dimensional force sensor installed at its end joint. Caregivers can use the robot arm’s teach pendant to adjust the applied force, as well as control the trajectory, speed, and duration of the robotic arm’s motion, ensuring maximum comfort for the elderly during bathing. The experimental setup captures key characteristics of real bathing interactions, including continuous contact, controllable force, and coordinated motion patterns, thereby providing a representative approximation of practical conditions. Consequently, the core challenge in enhancing comfort lies in the optimization of the objective function, which involves constructing an effective comfort model tailored to the scrubbing process.

### 2.2. Construction of a Tactile Somatosensory Comfort Model

Tactile sensation arises from physical stimuli, while tactile somatosensory comfort represents a comprehensive evaluation of the psychological and physiological responses induced by these stimuli [29]. Stevens’ law provides a framework to link physical stimuli to psychological response magnitudes, enabling the establishment of a relationship function between comfort and psychological responses through experimental analysis [30]. According to Stevens’ law, the rate at which psychological quantities increase depends on the type of stimulus, mathematically expressed as a power-law relationship: as perceptual experience increases arithmetically, the stimulus energy increases geometrically. Uniform descriptions of different physical stimuli can be achieved through energy transfer analysis [31]. In this study, physical stimuli are generated by the scrubbing tip, and the energy transfer between the scrubbing tip and the human body is calculated. By applying Stevens’ law to relate the energy-based physical stimulus to the psychological response, a tactile somatosensory comfort model is constructed. This section focuses on calculating scrubbing energy, integrating Stevens’ law, and building the comfort model based on the energy transfer function.

#### 2.2.1. A Model of the Relationship Between Physical Stimuli and the Amount of Psychological Response

The comfort experienced by the human body during the scrubbing process depends on various factors, including the physical stimulation applied to the skin. The level of stimulation is influenced by the characteristics of the human skin, the speed and amplitude of the scrubbing end, the contact force between the scrubbing end and the skin, and other parameters. The stimulation intensity can be quantified through the energy transfer between the scrubbing end and the human body during the scrubbing process, offering a measurable link between physical parameters and the comfort experienced.

The transfer of energy between the scrubbing end, which performs a single stroke, and the body is as follows:

(1) $$E=E_{f}+E_{e}$$

the motion of the scrubbing end combines rotational and translational movements around its own axis, allowing the energy transfer from the friction force to the human body to be decomposed into energy contributions from rotational motion and translational motion.

(2) $$E_{f}=E_{t}+E_{r}$$

(3) $$E_{t}=f_{n}\Delta S$$

classical friction theory posits that friction is directly proportional to normal pressure for rigid bodies. However, this principle does not adequately describe the relationship between skin friction and normal pressure. Comaish and Raja et al. propose that skin friction is instead proportional to an exponential function of the normal load:

(4) $$f_{n}=\mu N^{m}$$

the coefficient of friction *μ* between the skin and the scrubbing end, as well as the pressure *N* exerted between these surfaces, can be measured using a six-dimensional force sensor attached to the bathing robot arm. The relationship is described by a proportionality constant *m*, which typically ranges from 0.67 to 0.72 according to previous studies on skin friction and tactile perception [32]. In this study, m is set to 0.7, which lies within this commonly reported range and is adopted to ensure consistency with both literature and experimental conditions.

Assuming a disc with mass *m* as shown in Figure 2a, with radius *R* and friction coefficient *μ*, rotating uniformly on human skin. Consider a ring with radius *r* and infinitesimal width d*r* as a microelement. The total frictional moment of the disk can be calculated by means of differential integration: *M* = (2*μmgR*)/3. By substituting the gravitational force *mg* in the above equation with the positive pressure exerted by the scrubbing end, the resulting expression represents the frictional moment acting on the human body, denoted as *M* = (2*μN^m^R*)/3.

Therefore, the energy transfer between the friction force of the scrubbing end rotating through a single revolution and the skin is:

(5) $$E_{r}=\int_{0}^{2\pi }Md\theta =\frac{3\pi \mathrm{fr}}{4}$$

assuming that the bath robot arm moves the scrubbing end at a constant velocity, the scrubbing end rotates at a constant angular velocity, and it translates a linear distance of Δ*S*, the energy transfer during one revolution is given by:

(6) $$E_{f}=E_{t}+E_{r}=f\Delta S+\frac{4}{3}\pi \mathrm{fr}$$

assuming that the skin behaves as an elastomer, the energy transferred during one revolution of the scrubbing end to deform the skin consists of both normal deformation caused by pressure and tangential deformation induced by friction, i.e.,

(7) $$E_{\sigma }=\frac{1}{2}N\Delta l=\frac{1}{2}N\frac{Nl}{Y_{1}A}=\frac{N^{2}l}{2Y_{1}A}$$

similarly, the energy transferred due to the tangential deformation of the skin caused by one complete rotation of the scrubbing end can be expressed as:

(8) $$E_{\tau }=f\Delta s=f\frac{\tau l}{Y_{2}}=\frac{f^{2}l}{AY_{2}}=\frac{\left(\mu N^{m}\right)l}{AY_{2}}$$

since the bath robot arm can adjust the end position to ensure that the contact between the scrubbing end and the human body consistently aligns with the normal vector direction of the skin surface, *A* can be treated as the scrubbing end’s contact area during the bathing process. This area, *A = πr*^2^, remains constant.

(9) $$E=E_{f}+E_{\sigma }+E_{\tau }=E_{t}+E_{r}+E_{\sigma }+E_{\tau }=fS+\frac{4}{3}\pi fr+\frac{N^{2}l}{2Y_{1}A}+\frac{\left(\mu N^{m}\right)l}{AY_{2}}$$

the bathing time is denoted as Δ*t*, the translational distance of the scrubbing end is *S* = *v*Δ*t*, and the number of revolutions of the scrubbing end is *n* = (*ω*Δ*t*)/(2*π*). At this stage, the energy transfer between the scrubbing end and the human body can be expressed as:

(10) $$\begin{array}{ll} E & =fS+\frac{4}{3}\pi \mathrm{fr}+\frac{N^{2}l}{{2Y}_{1}A}+\frac{\left(\mu N^{m}\right)l}{{\mathrm{AY}}_{2}}\\ & =fv_{1}\Delta t+\frac{4}{3}\pi \mathrm{fr}\frac{\omega \Delta t}{2\pi }+\frac{N^{2}l}{{2Y}_{1}A}+\frac{\left(\mu N^{m}\right)l}{{\mathrm{AY}}_{2}}\\ & =(v_{1}+\frac{2}{3}v_{2})f\Delta t+\frac{N^{2}l}{{2Y}_{1}A}+\frac{\left(\mu N^{m}\right)l}{{\mathrm{AY}}_{2}} \end{array}$$

to simplify the energy function, most studies suggest that the energy absorbed by skin deformation constitutes only a small fraction of the total energy. Furthermore, due to the high elastic modulus of the skin, *E_σ_* and *E_τ_* are negligible within the scrubbing system. Additionally, to minimize the number of variables and simplify parameter fitting, *v*_1_ is assumed to equal *v*_2_. These assumptions are introduced to reduce model complexity and improve parameter identifiability, while preserving the dominant physical mechanisms governing tactile stimulation. Based on Equation (10), the total energy absorbed by the human body over a duration Δ*t*, considering only the stimulation caused by friction, can be expressed as:

(11) $$E=\frac{4}{3}vf\Delta t=\frac{4}{3}v\mu N^{m}\Delta t$$

although these simplifications may omit secondary effects such as nonlinear deformation and environmental interactions, experimental validation indicates that the resulting model retains sufficient accuracy for predicting tactile comfort under the studied conditions.

#### 2.2.2. Comfort Functions and Tactile Somatosensory Comfort Models

Stevens’ law posits that the psychological quantity *S* is a power function of the physical stimulus quantity *I*, expressed mathematically as *S* = *kI^n^*. Here, *S* represents the perceived intensity or sensory magnitude, *I* denotes the physical quantity of the stimulus, and *k* and *n* are constants specific to the type of sensory experience being rated. This implies that the psychological response is not logarithmic with respect to the stimulus quantity but follows a power function relationship [33]. However, Stevens’ law does not hold for weak stimuli near the perceptual threshold. To address this, Stevens and others proposed a modified power function in the early 1960s: *S* = *k*(*I* − *I*_0_)*^n^*. This adjustment incorporates a constant *I*_0_, which represents the absolute threshold value, making Stevens’ law applicable across the entire range of perceptible stimuli. Subtracting *I*_0_ from *I* effectively expresses the stimulus in terms of suprathreshold effective units rather than absolute physical units [34].

In this study, the relationship between the physical stimulus and the psychological response during scrubbing was established using Stevens’ law, under the following assumptions:

1. The external environmental stimulus quantity *I* is considered equivalent to the energy absorbed by the human body during the scrubbing process, *E*. This assumption is based on the physical interpretation that energy provides a unified measure of the combined effects of force, friction, velocity, and interaction duration, thereby serving as a comprehensive representation of stimulus intensity during scrubbing. While this formulation is primarily theoretically motivated, its validity is supported by the consistency between model predictions and both subjective perceptual ratings and physiological responses observed in the experiments.
2. The psychological response *S* is defined as the human body’s reaction to the stimulus during scrubbing.
3. The Stevens coefficient *n* depends on the type of stimulus experienced by the human body during scrubbing. For a given stimulus, *n* remains constant; however, when accounting for multiple stimuli such as friction and deformation, *n* represents the weighted influence of these different factors. In this study, the multi-stimulus interaction is first unified through the energy transfer formulation, and the exponent *n* is treated as an effective parameter identified through experimental fitting, which implicitly captures the combined perceptual sensitivity to different physical stimulus components. Rather than explicitly separating friction and deformation contributions, their joint effect is embedded in the fitted value of *n* under the given experimental conditions.
4. In the context of the scrubbing task, the stimulus arises solely from the contact between the scrubbing end and the human body, and thus *I*_0_ is assumed to be zero.

**Figure 3:**
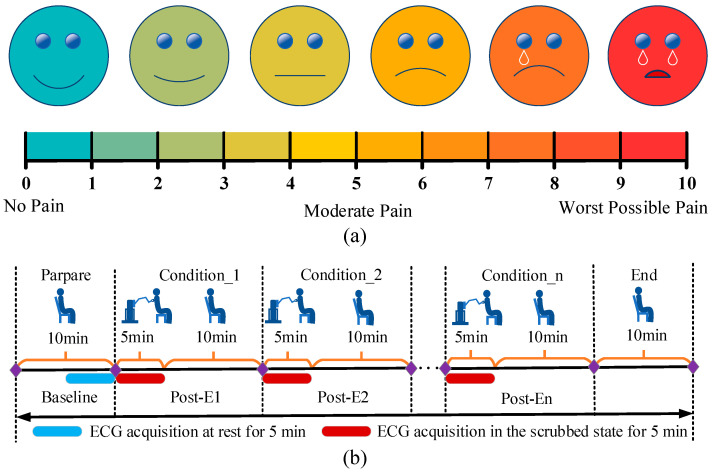
(**a**) Visual analogue scoring scale (VAS) and (**b**) experimental flow schematic.

Based on the simplified energy function (11), the functional relationship in Stevens’ law can be reformulated as follows:

(12) $$S=k{\left(\frac{4}{3}v\mu N^{m}\Delta t\right)}^{n}$$

by controlling various friction intensities through adjustments in speed and pressure, as outlined in Table 2, a visual analogue scoring scale (VAS) was used to quantify subjects’ subjective ratings of skin friction perception [35]. The VAS consisted of a 20 cm horizontal line printed on an A4-sized sheet, with markings at 0 for no sensation, 10 at the other extreme for severe pain, and intermediate sections divided into 2 cm intervals to indicate varying degrees of pain sensation, as shown in Figure 3a. The data were then fitted using Stevens’ power law formula *S* = *kI^n^* to determine the constants *k* and *n*.

The scrubbing end’s total running time *t* was set to 10 s, with 8 values assigned to both speed *v* and scrubbing pressure *N*, resulting in a total of 64 experimental conditions. Each volunteer completed 64 scrubbing experiments. To mitigate fatigue and reduce excessive skin friction caused by repetitive testing, five volunteers conducted experiments in batches at different time intervals, as shown in Table 3. Although full randomization of all experimental conditions was not implemented, the batch-wise design and time-separated sessions were adopted to reduce potential order effects such as fatigue and sensory adaptation. Future studies will incorporate randomized or counterbalanced experimental protocols to further minimize such effects. The study was approved by the Ethics Committee of Yixing Jiurucheng Rehabilitation Hospital (No. 20221104A08). Each subject signed a consent form. Experiments were carried out at the Laboratory of Shanghai Institute of Rehabilitation Engineering Technology. Comfort levels were calibrated using the VAS after each session. The experimental procedure is illustrated in Figure 3b. Specifically, each experiment followed a standardized protocol including subject preparation, parameter setting, controlled scrubbing execution, and post-experiment evaluation. Subjects were instructed to maintain a consistent posture, and scrubbing was performed along a predefined trajectory under controlled force and velocity conditions. Each trial was followed by subjective evaluation using the VAS scale and, where applicable, physiological data acquisition. The coefficient of friction *μ* for the nylon scrubbing tool was 0.25, and *m* was set to 0.7. The use of a single material (nylon) in all experiments ensures that the model fitting and validation focus on the influence of operational parameters (force and velocity), without additional variability introduced by different material properties.

The experimental scores were normalized, and the logarithmic form of Stevens’ power law was employed to analyze the relationship between physical stimuli and perceived sensation. The normalization process ensured consistency and comparability across different experimental conditions, while the logarithmic transformation provided a more linear relationship for data fitting. The results were used to calculate the parameters *k* and *n*, which represent the scaling constant and power index in Stevens’ law, respectively. This approach facilitated a precise evaluation of the psychological response to the physical stimulus.

**Figure 4:**
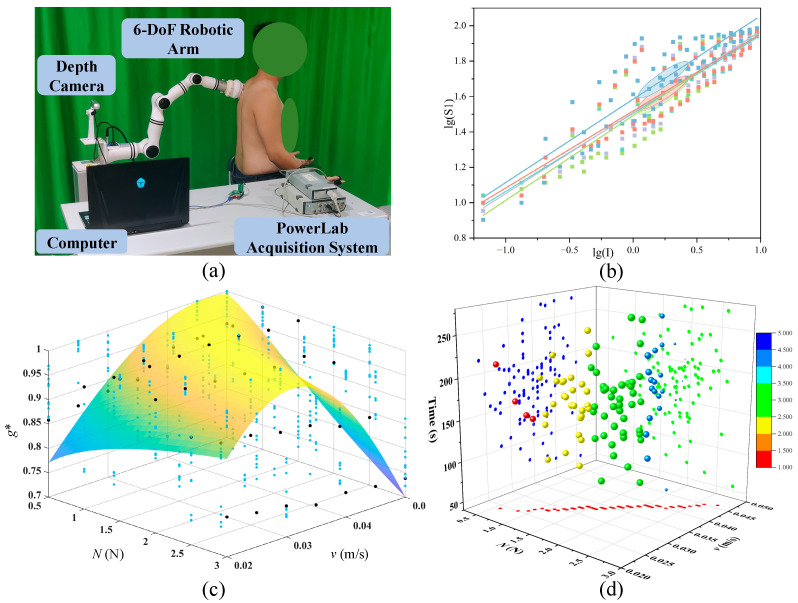
(**a**) Auxiliary scrubbing laboratory platform, (**b**) parameter fitting results, (**c**) nonlinear surface fitting based on the Physics-Informed Neural Networks (PINNs) algorithm and (**d**) optimal value solution based on particle swarm optimization algorithm.

(13) lg(S)=lg(k)+nlg(I)

the perceived intensity *S* and friction intensity *I* were subjected to logarithmic transformation, resulting in lg(*I*) and lg(*S*) data. These transformed data were analyzed using linear regression, where the slope *n* of the regression line represents the power exponent, and the intercept lg(*k*) is used to calculate the constant *k* as *k* = 10^lg(^*^k^*^)^. The experimental fit results, presented in Figure 4b, indicate a mean slope of *n* = 1.52 and an intercept of lg(*k*) = 0.46, yielding *n* = 1.52 and *k* = 2.88. The quality of the linear parameter fitting was evaluated using the coefficient of determination (R^2^) and the Sum of Squared Errors (SSE). The results indicate that the fitted model achieves a high R^2^ value close to 1 and a low SSE, demonstrating strong agreement between the fitted curve and the experimental data.

Considering the varying degrees of sensitivity of the human body to different physical stimuli, sensitivity indices *x* and *y* are introduced to the velocity *v* and pressure *N*. The higher the degree of sensitivity, the higher its power; conversely, the lower the degree of sensitivity, the lower its power. As a result, the Stevens’ law function is further extended to the following form:

(14) $$S=k{\left(\frac{4}{3}v^{x}\mu N^{my}\Delta t\right)}^{n}=0.54{\left(v^{x}N^{0.7y}\Delta t\right)}^{1.52}$$

during the scrub massage process, the physical stimulation of tactile perception, represented by the psychological parameter *S*, influences the subjective comfort experienced by the human body. It is necessary to establish a tactile perception comfort model to determine the relationship between the comfort value and the psychological parameter *S*.

In this study, a comfort function *g*(*S*) was developed, with *S* as the independent variable. Empirical evidence suggests that the relationship between the comfort function *g*(*S*) and *S* is not a simple monotonic one. Comfort is experienced when *k* falls within a specific range, whereas discomfort occurs when *k* is either too low or too high. This behavior exhibits distributional properties similar to those of the normal distribution function, expressed as follows:

(15) $$g\left(S\right)=\frac{1}{\sigma \sqrt{2\pi }}\exp\left(-\frac{{\left(S-\mu \right)}^{2}}{2\sigma^{2}}\right)$$

where *S* represents the tactile perceptual psychometric quantity, and *σ* and *μ* denote its variance and expected value, respectively.

The normal distribution function was modified to represent human comfort perception more accurately:

(16) $$g\left(S\right)=\frac{1}{\sigma \sqrt{2\pi }}\exp\left(-\frac{{\left(\frac{S}{b}-\mu \right)}^{a}}{2\sigma^{2}}\right)$$

where *a* and *b* are parameters that regulate the decay rate of the function curve, thereby shaping the curve’s overall form.

The comfort function *g*(*S*) is defined with the following conditions: (1) its maximum value is 1; and (2) its initial value is 0.5, indicating that in the absence of external stimuli and tactile perception (i.e., when *S* = 0), the comfort level is 0.5. This value is introduced to represent a neutral baseline state rather than either maximal comfort or explicit discomfort. In other words, when no additional scrubbing-induced tactile stimulus is present, the human body is assumed to remain in an intermediate comfort condition, from which comfort may increase under appropriate stimulation or decrease under insufficient or excessive stimulation. This setting is therefore a normalized and conceptually motivated modeling assumption used to characterize the non-monotonic nature of comfort perception.

Based on the above assumptions, we derive $\sigma =\frac{1}{\sqrt{2\pi }}$ and $u={\left(\frac{\ln2}{\pi }\right)}^{\frac{1}{a}}$. Substituting these into Equation (10) yields:

(17) $$g\left(S\right)=\exp\left(-\pi {\left(\frac{S}{b}-{\left(\frac{\ln2}{\pi }\right)}^{\frac{1}{a}}\right)}^{a}\right)$$

by associating Equation (14) with Equation (17), the comfort perception optimization model *g*(*S*) is derived as follows:

(18) $$\begin{array}{ll} g\left(S\right) & =\exp\left(-\pi {\left(\frac{k}{b}{\left(\frac{4}{3}v^{x}\mu N^{\mathrm{my}}\Delta t\right)}^{n}-{\left(\frac{\ln2}{\pi }\right)}^{\frac{1}{a}}\right)}^{a}\right)\\ & =\exp\left(-\pi {\left(\frac{0.54}{b}{\left(v^{x}N^{0.7y}\Delta t\right)}^{1.52}-{\left(\frac{\ln2}{\pi }\right)}^{\frac{1}{a}}\right)}^{a}\right) \end{array}$$

the parameters to be determined in Equation (18), *x* and *y*, are derived through experimental fitting and data analysis. Equation (18) demonstrates that human comfort perception is primarily influenced by the robot’s operating speed, the normal load, and the sensitivity coefficient of the human body to these operating parameters.

### 2.3. Comfort-Awareness-Based Fitting of Robot Operating Parameters

Human comfort perception varies due to individual differences, particularly when the robot performs scrubbing operations on different body areas across various individuals. Factors such as gender, age, height, weight, and skin sensitivity significantly influence human comfort perception [36]. Therefore, optimizing the robot’s operating parameters requires considering individual variability in perception and the factors influencing comfort, to enhance both human comfort and the robot’s operational efficiency.

#### 2.3.1. A Model of the Relationship Between Physical Stimuli and the Amount of Psychological Response

To parameterize the comfort model, a scrubbing experimental platform was designed to simulate the robotic bath-assisted scrubbing process. The experimental setup consists of a 6-DoF robotic arm, a scrubbing end-effector, a binocular depth camera, and a computer for data acquisition and processing. A 6-dimensional force sensor mounted at the end of the robotic arm collects real-time six-axis force data during the experiment, enabling precise control of the contact force between the robotic arm and the human body. The scrubbing end-effector comprises a drive mechanism and a detachable brush head. The scrubbing end-effector supports 360° automatic rotation and allows for quick and easy removal and replacement of the brush head. The brush head used in this study is made of nylon. The schematic diagram of the experimental setup is shown in Figure 4a.

#### 2.3.2. Experimental Design

The comfort perception experiment investigates the influence of various robot operating parameters on human tactile comfort perception under diverse experimental conditions, and the experimental data are used to fit the parameters of the comfort perception optimization model, providing a foundation for optimizing the operating parameters of the assisted bathing robot.

The tactile comfort perception of test subjects is influenced by factors such as gender, weight, and height [37]. Differences in skin structure among subjects with varying genders, weights, and heights result in distinct psychological responses to comfort perception during scrubbing [38]. In the comfort perception experiment, it is essential to control variables such as age, gender, height, and weight of the test subjects, while maintaining consistent arm skin treatment conditions [39]. Ten healthy adult volunteers participated in the experimental protocol. Their personal data are summarized in Table 4. In total, 20 participants (10 males and 10 females) were included in this study, which falls within the typical range (10–30 participants) reported in related tactile perception and human–robot interaction studies. This sample size is considered adequate for controlled experimental validation, although larger-scale studies will be conducted in future work to further improve generalizability. The participants were selected within a relatively controlled range of age, height, and weight to reduce inter-subject variability; however, this cohort does not fully represent broader populations with diverse demographic and physiological characteristics (e.g., elderly individuals, different skin types, or clinical conditions). The study was approved by the Ethics Committee of Yixing Jiurucheng Rehabilitation Hospital (No. 20221104A08). Each subject signed a consent form. Experiments were carried out at the Laboratory of Shanghai Institute of Rehabilitation Engineering Technology. For each volunteer, the palmar skin of both the left and right forearms was tested. The current experimental validation is therefore based on specific body regions (forearm and back), and the identified model parameters are calibrated for these regions under controlled conditions. To ensure the consistency of the experiment, scrubbing was performed along a fixed trajectory, moving back and forth three times following the midline of the human back spine, as shown in Figure 2b. Each experimental condition (combination of force and velocity) was applied under controlled settings, with consistent motion duration and predefined parameter ranges. Subjects were given sufficient rest intervals between trials to minimize fatigue and sensory adaptation. All volunteers were instructed to refrain from using any chemical or cosmetic substances on the test site the day prior to the experiment to maintain the skin’s natural state. In addition, experiments were conducted under controlled environmental conditions with standardized posture and rest intervals to reduce variability. However, baseline physiological factors such as stress levels, hydration status, and circadian rhythm were not strictly quantified, which may influence physiological and perceptual responses. The test area was first washed with regular shampoo, rinsed thoroughly with water, dried using a lint-free towel, and subsequently cleaned with alcohol. Volunteers were also asked to abstain from physical exercise for at least 15 min before each scrubbing and physiological test.

Based on feedback from pre-experimental volunteers, who determined the constant *k* and power index *n*, the normal load *N* was found to be most comfortable within the range of 0.5 N to 3 N, while the end scrubbing speed ranged between 20 mm/s and 50 mm/s. Consequently, the normal contact force *N* during the experiment was set to range from 0.5 N to 2.5 N, with an increment of 0.5 N. The end scrubbing speed *v* was configured to range from 25 mm/s to 55 mm/s, with an increment of 5 mm/s. The experimental conditions are shown in Table 5.

During the experiments, the robot scrubbed the forearm along its horizontal length under predefined conditions. To minimize fatigue and skin friction from repeated experiments, testers performed the experiments in separate time intervals and provided comfort ratings based on their subjective perceptions. Given the individual differences in stimulus perception, the comfort ratings were normalized to ensure an accurate assessment of testers’ tactile perceptions of the scrubbing stimuli.

Experimental data points (*N*, *v*, *g*), comprising the normal contact force (*N*), end scrubbing speed (*v*), and arm skin comfort rating (*g*), were collected after the comfort experiment.

#### 2.3.3. Data Analysis and Fitting of Model Parameters

The comfort perception optimization model is determined by fitting its parameters using the results from the comfort perception experiments. An approximation function *gψ* is constructed using the *m* × *v* × *n* dataset obtained from the experiments. To enhance the accuracy of the approximation of *gψ* to the comfort model *g* within the range of experimental data, a fitting function is developed using a nonlinear surface fitting method based on the Physics-Informed Neural Networks (PINNs) algorithm [40]. Compared with conventional nonlinear regression or purely data-driven machine learning methods, PINNs incorporate physical constraints derived from the underlying model into the learning process, thereby ensuring consistency with the energy-based formulation and improving generalization under limited data conditions. This approach enables a balance between physical interpretability and data fitting capability, making it particularly suitable for modeling tactile comfort in this study. This approach is adopted not only for accurate fitting, but also to incorporate the physical constraints embedded in the derived comfort model, thereby improving parameter identifiability, interpretability, and robustness under limited experimental data. The fitting curve is constructed as follows:

(19) $$R-\mathrm{square}=\sum_{i=1}^{N}{\left(g_{\psi_{i}}-g_{i}\right)}^{2}$$

during the fitting process, to accurately capture the overall shape of the comfort function *g*(*S*), an objective function for minimizing the error is derived. The goal is to ensure that the Sum of Squared Errors (SSE) is minimized and the coefficient of determination (*R*^2^) is as close to 1 as possible, indicating a better fit. In this study, the fitted model achieved an SSE value of 0.0068 and an R^2^ value of 0.982, demonstrating a high level of agreement between the predicted results and the experimental data. Using this approach, the parameters *x*, *y*, *a*, and *b* are determined to construct the scrubbing comfort model.

(20) $$g^{*}=\exp\left(-\pi {\left(\frac{0.54}{b}{\left(v^{x}N^{0.7y}\Delta t\right)}^{1.52}-{\left(\frac{\ln2}{\pi }\right)}^{\frac{1}{a}}\right)}^{a}\right)$$

where *x* and *y* represent the sensitivity indices for the operating speed (*v*) and the normal load (*N*), respectively, while *a* and *b* are the correction coefficients of the comfort function, which is aligned to the normal distribution.

The PINNs algorithm was applied to fit the constructed comfort model (Equation (21)) using experimental data on subjects’ comfort perception in the arm region, as illustrated in Figure 4c. Although simpler fitting approaches (e.g., polynomial or exponential regression) could also be used for data approximation, they lack physical interpretability and may exhibit limited extrapolation capability. In contrast, the PINNs-based approach leverages both data and physical constraints, resulting in a more reliable and physically consistent model. A quantitative comparison with alternative models will be explored in future work. The fitted experimental data yielded sensitivity indices *x* = 1.66 and *y* = 3.87 for *v* and *N*, respectively, along with curve form factors *a* = 1.04 and *b* = 2.01. The magnitude of the sensitivity indices indicates that the human body is more sensitive to the robot’s normal load than to its operating speed.

## 3. Results

### 3.1. Optimization of Model Operating Parameters

#### 3.1.1. Objective Function Construction

To ensure that the human body’s perceived comfort evaluation value g*, under varying normal loads (*N*) and operating speeds (*v*), aligns as closely as possible with the “comfort” comfort evaluation value *G*, the optimization objective function is formulated using Equation (21):

(21) $$G\left(v,N\right)=G-\exp\left(-\pi {\left(2.51{\left(\frac{4}{3}v^{x}\mu N^{0.7y}\Delta t\right)}^{1.53}-{\left(\frac{\ln2}{\pi }\right)}^{\frac{1}{2}}\right)}^{2}\right)$$

#### 3.1.2. Optimization of Parameter Settings

Using the particle swarm optimization algorithm, the objective function is minimized to determine the optimal values for the normal load and operating speed parameters. Since this robot scrubbing massage experiment pertains to general body cleaning without medical rehabilitation or specialized operational requirements, the scrubbing massage robot operational parameters were set using the optimization mathematical model (Equation (21)) as outlined in Table 6.

#### 3.1.3. Optimization Results

The parameters of the established tactile body comfort model are optimized using the Particle Swarm Optimization (PSO) algorithm [41], with the distribution space illustrated shown in Figure 4d. PSO is selected due to its effectiveness in solving nonlinear and non-convex optimization problems without requiring gradient information. Compared with gradient-based methods, it avoids issues related to local minima and gradient computation, and compared with evolutionary algorithms such as Genetic Algorithms, it offers a simpler structure and faster convergence in continuous parameter spaces. This makes it particularly suitable for optimizing the comfort model in this study. The optimized results for the robot operating parameters are presented in Table 7.

### 3.2. Experimental Verification

To verify the comfort of the optimal scrubbing parameters (force *N* = 1.68 N and velocity *v* = 36.47 mm/s) obtained through model fitting, 20 subjects (10 males and 10 females) were recruited, and their arms were scrubbed and massaged under identical experimental conditions. The experiment lasted for 5 min, during which heart rate variability (HRV) parameters, including the standard deviation of all normal-to-normal R-R intervals (SDNN), Root mean square of successive differences (RMSSD), Percentage of intervals different by more than 50 ms from preceding interval (pNN50), High frequency power normalized (HFnorm), Low frequency power normalized (LFnorm) and Low Frequency to High Frequency Ratio (LF/HF), were continuously monitored in real time.

**Figure 5:**
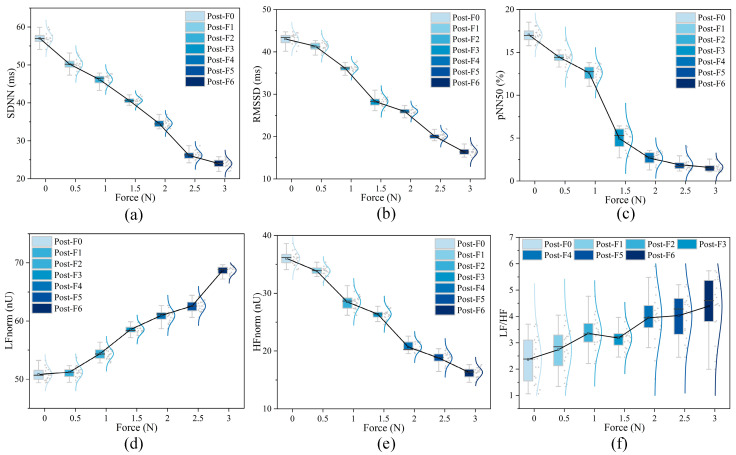
(**a**) SDNN, (**b**) RMSSD, (**c**) pNN50, (**d**) HFnorm, (**e**) LFnorm, and (**f**) LF/HF results under different contact force conditions.

**Figure 6:**
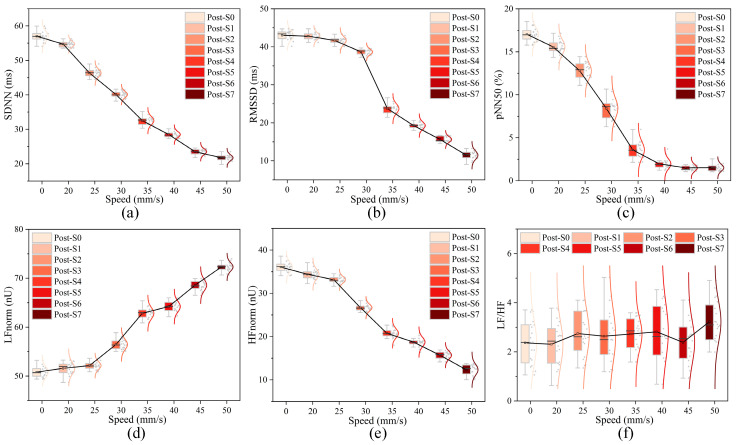
(**a**) SDNN, (**b**) RMSSD, (**c**) pNN50, (**d**) HFnorm, (**e**) LFnorm, and (**f**) LF/HF results at different scrubbing speed conditions.

The experimental results are shown in Figure 5 and Figure 6. Under the optimized parameters, the subjects’ HRV parameters demonstrated enhanced parasympathetic activity, evidenced by a significant increase in RMSSD, pNN50, and HFnorm, as well as a significant decrease in LF/HF, reflecting a more relaxed mental state. Both male and female subjects achieved the highest comfort scores under these conditions, with minimal differences in HRV parameter changes. This result demonstrated that the scrubbing parameters derived from model optimization had universal applicability, significant comfort effects, and provided robust experimental support for the optimal design of tactile comfort. It should be noted that HRV primarily reflects autonomic nervous system activity, particularly parasympathetic regulation, and is used in this study as a supportive physiological indicator rather than a direct or exclusive measure of subjective comfort.

## 4. Discussion

This study developed a mathematical model of tactile somatosensory comfort by integrating Stevens’ law and the energy transfer function, and validated the model using a scrubbing massage robot. In particular, the assumption that stimulus intensity can be represented by energy transfer (*I* = *E*) provides a physically consistent and computationally tractable formulation; however, it remains an approximation that may not fully capture all aspects of human tactile perception, especially under complex or dynamic real-world conditions. Future work will explore more detailed representations of stimulus characteristics, including temporal and spatial effects. The optimal massage speed and intensity were identified through an optimization algorithm. Moderate scrubbing parameters were shown to significantly enhance physiological comfort, as evidenced by increased parasympathetic activity (elevated RMSSD and HFnorm) and moderately balanced sympathetic activity (reduced LFnorm and LF/HF). These findings suggest that higher somatosensory comfort can be achieved by enhancing autonomic balance through a specific combination of massage speed and intensity, offering a scientific foundation for the optimal design of scrub massage robots.

Compared to previous studies, this study demonstrates progress in several key areas. Notably, this study is the first to integrate Stevens’ law with the energy transfer function to develop a tactile somatosensory comfort model. It fills the gap in tactile comfort research oriented towards quantitative modelling of weak stimuli. In contrast to prior research methods, which primarily relied on empirical or single-variable analyses, this model systematically characterizes the relationship between force and speed on comfort during scrubbing and massage by incorporating the energy transfer mechanism, while accurately capturing the nonlinear characteristics of perceived intensity using Stevens’ law. While previous studies have demonstrated that moderate massage force and rhythm can activate parasympathetic nerves, resulting in a sense of comfort [37], this paper further quantifies the relationship between force and speed through mathematical modelling and optimization algorithms. For the first time, it reveals differences in heart rate variability (HRV) metrics under varying scrubbing forces and speeds. Specifically, scrubbing speed exerts a greater influence on parasympathetic indicators such as RMSSD and pNN50, whereas scrubbing force has a stronger impact on sympathetic indicators like LF and LF/HF. This quantification of the differential effects of scrubbing parameters offers theoretical support for the development of personalized scrubbing parameter designs.

Although this study yielded valuable findings, it has certain limitations. First, the small sample size (10 males and 10 females) may limit the generalizability of the findings and may not fully capture inter-individual variability in tactile perception. In addition, the participant cohort is relatively homogeneous in terms of age and health condition, and does not fully capture variability in skin properties, physiological states, or clinical populations, which may influence tactile perception and comfort evaluation. Future studies will include more diverse participant groups to improve the robustness and applicability of the proposed model. Second, the experimental conditions were relatively uniform, focusing solely on combinations of scrubbing strength and speed, and were conducted under controlled laboratory conditions, which may not fully represent the complexity of real-world assistive care environments. In particular, real bathing scenarios may involve additional factors such as water flow, skin wetness, temperature variations, and dynamic human movement, which were not fully incorporated in the current experimental design. Therefore, the present study should be regarded as a controlled yet representative approximation of real-world conditions. Future work will focus on incorporating these factors to further enhance ecological validity. Additionally, variations in individual physiological and psychological states (e.g., stress levels and emotional conditions) could have influenced the experimental results, but these factors were not fully controlled in this study. Furthermore, the current experimental design is limited to a specific interaction task and parameter space, and does not yet account for more complex tactile patterns or multimodal environmental influences. Future work will incorporate randomized or counterbalanced designs to improve experimental rigor. Finally, changes in individual physiological parameters may have been affected by external environmental factors, and their capacity to independently reflect psychological relaxation still requires further validation through multimodal and large-scale studies. Future work will focus on relaxing these assumptions and incorporating additional physical and perceptual factors to enhance model generalizability.

This study proposes a tactile somatosensory comfort modeling method, exemplified by human back scrubbing performed by our bathing assistive robot. The method described in this paper is applicable to evaluating the comfort of various devices interacting with the human body, demonstrating its universal applicability. However, due to variations in skin properties across different anatomical regions (e.g., thickness, elasticity, and sensitivity), the model parameters may require region-specific calibration to maintain predictive accuracy. Future work will focus on extending the model to multiple body regions and developing adaptive parameterization strategies to improve generalizability. Future research can be expanded in several key directions. First, increasing the sample size and including a broader range of subjects (e.g., diverse age groups and occupational backgrounds) can enhance the generalizability of the findings. Second, expanding experimental conditions to explore the combined effects of various tactile modalities and environmental factors on comfort is essential. Additionally, HRV results can be cross-validated with multidimensional indicators, such as subjective scores and brainwave data (EEG), to improve the reliability and interpretive depth of the findings [38]. With advancements in technology, intelligent robots capable of real-time adjustment of massage parameters can be developed to optimize the tactile experience through dynamic regulation. With these improvements and extensions, research on tactile somatosensory comfort is poised to achieve significant breakthroughs in healthcare, wellness, and human-computer interaction.

## 5. Conclusions

This study proposes a mathematical model of tactile somatosensory comfort based on Stevens’ law and the energy transfer function, with its validity verified through scrubbing massage robot design experiments. An optimization algorithm was employed to determine the optimal scrubbing speed and strength, with experimental results demonstrating that moderate scrubbing parameters effectively enhance physiological comfort. Additionally, the study examines the effects of varying scrubbing forces and speeds on heart rate variability (HRV) parameters, providing data-driven insights for optimizing personalized tactile experiences. By integrating mathematical modelling with multi-dimensional physiological data analysis, this study significantly advances the quantitative understanding of tactile comfort and provides a scientific foundation for deploying scrubbing and massage robots in healthcare and recreational contexts. Future research can enhance the model’s robustness and applicability by increasing the sample size, diversifying experimental conditions, and developing intelligent regulation systems to accommodate diverse scenarios and foster the widespread adoption of tactile technology.

## Figures and Tables

**Table 1:** List of mathematical notations.

Notation (Unit)	Definition & Typical Values
*E* (J)	The total energy transferred (1 × 10^−3^–1 J)
*E_f_* (J)	The heat generated from friction (1 × 10^−4^–1 × 10^−2^ J)
*E_e_* (J)	The energy associated with skin deformation (1 × 10^−3^–5 × 10^−3^ J)
*E_t_*, *E_r_* (J)	The energy transferred by the frictional force during the rotational and translational motion of the scrubbing end (1 × 10^−4^–1 × 10^−2^ J)
Δ*S*, *f* (mm, N)	The displacement of the scrubbing end, the frictional force (0–100 mm, 0.1–5 N)
*μ*, *N* (/, N)	The coefficient of friction, the pressure exerted (0.2–0.5, 5–20 N)
*R*, *r* (mm)	The scrubbing end radius, radius of a rotating microelement (50 mm, 0–50 mm)
*M* (N/m)	The total frictional moment on the entire disc (1 × 10^−4^–5 × 10^−4^ N/m)
*E_σ_*, *E_τ_* (J)	The energy transferred due to the normal and tangential deformation of the skin (1 × 10^−3^–5 × 10^−3^ J)
*l* (mm)	The thickness of the skin and muscle tissue (5–10 mm)
*A* (mm^2^)	The contact area between the skin and the scrubbing end (7850 mm^2^)
*Y*_1_, *Y*_2_ (Pa)	The normal and tangential modulus of elasticity of the skin (5 × 10^4^–2 × 10^5^ Pa, 1 × 10^4^–×10^5^ Pa)
*S*, *τ* (Pa)	The perceived intensity or sensory magnitude, the shear stress (1–10, 5 × 10^2^–2 × 10^3^ Pa)
*v*_1_, *v*_2_ (mm/s, mm/s)	The speed of translation and rotation of the scrubbing end (10–80 mm/s, 10–80 mm/s)
*I*, *I*_0_ (J)	The physical quantity of the stimulus (1 × 10^−3^–1 J, 1 × 10^−3^–1 J)
*k*, *n* (/)	Constants specific to the type of sensory experience being rated (0.3–1, 1–3)

**Table 2:** Experimental parameter setting.

***t*** (s)	10							
***N*** (N)	0.1	0.5	1	2	3	4	5	6
***v*** (mm/s)	10	20	30	40	50	60	70	80

**Table 3:** Pre-experimental Volunteer profile.

Number	Gender	Age (Year)	Weight (kg)	Height (cm)
M1	Male	58	67.3	171.4
M2	Male	62	65.2	173.6
M3	Male	63	73.1	178.5
F1	Female	59	57.5	165.6
F2	Female	61	53.6	162.3

**Table 4:** Volunteer profile.

Gender	Number	Age (Year)	Weight (kg)	Height (cm)
Male	5	25 ± 3	65.2 ± 5	171.5 ± 4
Female	5	25 ± 2	52.6 ± 4	162.7 ± 3

**Table 5:** Experimental parameter setting.

***N*** (N)	0.5	1	1.5	2	2.5	3	
***v*** (mm/s)	20	25	30	35	40	45	50

**Table 6:** Optimization of parameter settings.

Optimization of Parameter Settings	
Objective function	Min G (*v*, *N*)
	G = 1
Restrictive condition	0.5 N ≤ *N* ≤ 3.0 N
	0.02 mm/s ≤ *v* ≤ 0.05 mm/s

**Table 7:** Optimization results.

G (*v*, *N*)	*v*	*N*
0.0018	*v* = 36.47 mm/s	1.68 N

## Data Availability

The original contributions presented in this study are included in the article material. Further inquiries can be directed to the corresponding author.
